# The evolving landscape of expanded carrier screening: challenges and opportunities

**DOI:** 10.1038/s41436-018-0273-4

**Published:** 2018-09-24

**Authors:** Stephanie A. Kraft, Devan Duenas, Benjamin S. Wilfond, Katrina A. B. Goddard

**Affiliations:** 1 Treuman Katz Center for Pediatric Bioethics, Seattle Children’s Hospital and Research Institute, Seattle, WA USA; 2 Division of Bioethics and Palliative Care, Department of Pediatrics, University of Washington School of Medicine, Seattle, WA USA; 3 Center for Health Research, Kaiser Permanente Northwest, Portland, OR USA

**Keywords:** pan-ethnic screening, clinical genomics, ethical, legal, and social implications

## Abstract

Carrier screening allows individuals to learn their chance of passing on an autosomal or X-linked condition to their offspring. Initially introduced as single-disease, ancestry-based screening, technological advances now allow for the possibility of multi-disease, pan-ethnic carrier screening, which we refer to as “expanded carrier screening.” There are numerous potential benefits to expanded carrier screening, including maximizing the opportunity for couples to make autonomous reproductive decisions, and efficiency and marginal additional costs of including more conditions if the test is already being offered. While numerous laboratories currently offer expanded carrier screening services, it is not yet commonly used in clinical practice, and there is a lack of consensus among experts about the service, including whether this should be offered to individuals and couples, whether this should be offered preconception or prenatally, and what conditions to include in screening programs. Challenges for expanded carrier screening programs include a lack of demand from the public, low prioritization by health systems, the potential for pressure to undergo screening, the possibility of disability-based discrimination, needed adaptations to pre- and post-test counseling, technical limitations, and the evolving technological and socio-political landscape.

## Introduction

Carrier screening programs were introduced in the 1970s to offer individuals the opportunity to learn the likelihood that they could pass on an autosomal or X-linked condition to their offspring. Initially, carrier screening programs were used only with ethnic groups who had relatively high incidence of certain conditions, such as ancestry-based screening for Tay–Sachs disease in Ashkenazi Jewish communities and β-thalassemia in Mediterranean populations.^1,2^ After the 1989 identification of the gene associated with cystic fibrosis (CF), CF screening became available,^3,4^ and by the late 1990s the professional consensus was that it should be offered to all individuals or couples seeking prenatal or preconception care.^5^ Subsequently, several professional guidance documents have been issued in support of pan-ethnic CF screening.^6–8^ This move has been motivated in part by the difficulty of assigning individuals to a single ethnicity and determining who is at risk for ancestry-based screening, and to provide more equitable access to carrier screening services. Throughout the past decade, it has become increasingly common to offer carrier screening to the general preconception and prenatal populations.^9–12^ For example, in some parts of the world, pan-ethnic screening is common for hemoglobinopathies and thalassemias,^13^ and it has recently been recommended for conditions such as spinal muscular atrophy^14–16^ and fragile X syndrome.^16^

In addition to the increase in pan-ethnic screening, newly efficient genetic technologies, such as next-generation sequencing, are a driving force behind the expansion of carrier screening to more conditions. This technology has allowed for expanded carrier screening panels that include hundreds of conditions.^17–20^ While many conditions are rare individually, Srinivasan et al.^21^ found that about 35% of individuals in their sample were carriers of at least one condition, leading them to conclude that “screening for the most common genetic diseases alone will fail to discover most of the carriers in the general population.”

There are several potential benefits of identifying carriers of more conditions and across more populations. First, including more conditions and more people in carrier screening programs could maximize the opportunity for couples to make autonomous reproductive decisions. Second, it could normalize carrier status and thus reduce the risk of social stigmatization. Finally, expanded carrier screening may be more cost-effective than single-disease, ancestry-based testing.^18,20^ For expanded carrier screening to become an increasingly common way for parents and prospective parents to learn about their genetic risk, we must understand what implications this technology has for patients, clinicians, health-care systems, and society. Despite its potential advantages, there are also potential adverse consequences and challenges to implementation of these programs. In this paper, we report on the current landscape, identify potential challenges that should be addressed, and describe future opportunities for use of expanded carrier screening as a clinical service.

## The current landscape of expanded carrier screening

### Screening availability and utilization

Chokoshvili et al.^22^ reported that in 2017, 16 providers offered expanded carrier screening, 13 of which were commercial companies. Panels included as few as 41 conditions and as many as 1556, with most providers offering screening for between 100 and 300 conditions. In most cases, screening is offered through a physician-moderated model, whereby the company markets the test directly to the public but requires it to be ordered by a physician, or directly embedded within clinical care.^12,23,24^

While data that track the number of individuals who undergo carrier screening are not publicly available, Haque et al.^25^ reported that over 400,000 individuals received expanded carrier screening through one laboratory between 2012 and 2015. Lazarin and Haque^26^ subsequently estimated that over 200,000 individuals in the United States, as compared with 4 million annual pregnancies, received expanded carrier screening in 2015.

### Limited clinician experience

Studies of clinicians’ views on expanded carrier screening have revealed that many have limited clinical experience with it. Lazarin et al.,^27^ for example, surveyed 337 genetic counselors in the United States and Canada and found general support for expanded carrier screening and an expectation that it would become routine practice, but most were not offering it clinically at the time of the survey in 2012. Also in 2012, Benn et al.^28^ surveyed 222 practicing obstetrician/gynecologists in the United States about their use of expanded carrier screening. Of the respondents, 15% said they offered it to all of their patients and just over half offered it on request, while 40% believed carrier screening should be restricted to individuals at high risk of being a carrier. There is a need for more current data regarding clinicians’ familiarity with this service because it has become more common over the past several years. Such data can identify needs for clinical supports, including professional education, for those who interact directly with patients who are deciding whether to undergo screening and how to interpret and act on results. This is especially important when individuals become aware of expanded carrier screening through direct-to-consumer marketing and physicians serve as moderators between test companies and those considering screening.

### Professional guidance

Professional societies for genetics and/or reproductive medicine have issued guidance documents on the use of expanded carrier screening. These guidance documents address three primary considerations: who should be offered expanded carrier screening, what the preferred timing is for screening, and what conditions should be included on screening panels.

#### Should expanded carrier screening focus on couples or individuals?

Most professional societies recommend offering expanded carrier screening to all women and their partners who are planning a pregnancy, regardless of ancestry.^9,15,29–31^ This pan-ethnic approach differs from a universal, or population-wide, approach because it targets the subpopulation of individuals and couples who are in the prenatal or preconception period, rather than all individuals. Some providers have questioned whether expanded carrier screening should be offered only to couples, or whether results should be disclosed to individuals.^32^ Offering screening only to couples provides the greatest clinical utility and public health benefit, and would reduce the workload for clinicians compared with reporting out individual results, because few couples are carriers of the same autosomal recessive conditions. This approach would also ensure that couples decide together and agree upon expanded carrier screening. However, individual carrier screening still allows for cascade screening to family members and can reduce uncertainty and worry for individuals with a known familial risk as well as enable them to use the information if they change partners.

In one study that took an individual preconception carrier screening approach, 102/131 (76%) women were carriers for at least one condition. Of those women’s male partners, 71/102 (70%) chose to participate and were subsequently screened.^33^ Only 6% of women declined to receive carrier screening in this program because of partner resistance.^34^ Plantinga et al.^35^ assessed public perspectives about expanded carrier screening being offered only to couples among 504 Dutch couples of reproductive age and found that 70% did not object to a couple-only approach, 15% objected, and the remainder were undecided. Holtkamp et al.^36^ explored this question with a Dutch Jewish population, which may have had more exposure to carrier screening from ancestry-based programs. In this population, 46% preferred only to be informed as a couple, while 39% preferred full disclosure of the individual test results.

#### What is the preferred timing for screening?

There is widespread consensus among professional societies and most commenters that the ideal time to offer carrier screening is preconception, as this allows for more options (e.g., avoiding pregnancy or using advanced reproductive technologies to conceive) than does screening during a pregnancy.^9,15,31^ Additionally, current guidance documents suggest that screening of both individuals in a couple may take place either sequentially or concurrently, but concurrent screening may be recommended if there are time constraints that could limit the reproductive actionability of the results—for example, if screening is done during a pregnancy.^30^

While preconception care is widely recognized as an important service,^37,38^ most women do not receive these services currently. In the United States, data from selected states indicate that only 33% of women with a recent live birth had a discussion with a health-care professional prior to their pregnancy about improving their health.^39,40^ A review of CF carrier screening studies found lower uptake of screening in the preconception setting than in prenatal care despite a general belief that preconception is the ideal timing, which the authors attributed to many individuals’ lack of interest in screening prior to conception.^4^ For expanded carrier screening to be delivered as part of preconception care, couples would need to approach planning for pregnancy differently.

#### What conditions should be included on screening panels?

Current guidance documents do not specify which conditions should be included on an expanded panel, but most recommend at least some specific conditions such as CF and spinal muscular atrophy.^14–16^ There is also general consensus among professional societies that expanded carrier screening panels should focus on childhood-onset conditions that are likely to have a significant impact on the child’s quality of life.^9,15,31,41,42^ In addition to age of onset and clinical impact, most guidance documents also include criteria related to the scope of the condition (including frequency of the gene and penetrance of the phenotype) and the extent to which parents and/or providers can take action in response to a positive finding. However, professional societies vary in terms of the specificity of their lists of considerations and/or criteria, as well as the details of their guidance. Table 1 shows the range of considerations included among these documents.

**Table 1:** Categories of considerations for including a gene/condition on expanded carrier screening panels

Categories	Examples of considerations
**Scope**	• Frequency of gene • Penetrance of phenotype • Prevalence of condition
**Impact**	• Effect on quality of life • Degree of cognitive or physical impairment • Need for surgical or medical intervention
**Age of onset**	• Childhood vs. adult onset
**Actionability**	• Pertinence to reproductive decision making • Availability of prenatal diagnosis • Availability of prenatal intervention, delivery management, or prenatal education regarding special needs

Defining the impact of conditions as “severe” or “serious” often proves challenging.^43^ Lazarin et al.^44^ developed a systematic classification of disease severity, arguing that severity is a good criterion because couples do not alter their plans when the condition is perceived as mild, and providers may refuse steps to prevent the birth of an affected child with a mild condition on moral or legal grounds. Some professionals and laypersons may have similar views on severity,^45,46^ but other clinicians have objected to classifications based on severity.^47^

In terms of patient and public preferences for conditions to include in expanded carrier screening, Holtkamp et al.^36^ surveyed 145 Dutch individuals with Ashkenazi Jewish ancestry and found that 37% preferred an expanded panel containing a closed list of diseases, while 43% thought people should be able to decide for themselves in what categories of disorders they wanted to be tested. Kraft et al.^48^ found that presenting categories of conditions can support patient decision making. The authors interviewed 51 individuals who were enrolled in a randomized study of genomic-based carrier screening during the preconception period and found that, while most of them chose to receive all categories of results (including conditions in adult onset, variable, severe, mild, and lifespan limiting categories), they also valued the opportunity to select from several different categories of results. Using a taxonomy of conditions may simplify the decision-making process and potentially minimize the complexity of consent to screening for a broad range of conditions with a variety of implications.^13^

## Challenges for expanded carrier screening

### The general public may lack interest in expanded carrier screening

Lack of public interest in expanded carrier screening is related to both awareness and knowledge.^49^ Compared with ancestry-based high-risk groups, the general population is less familiar with genetic conditions and has less knowledge of or experience with families with an affected child. It is unclear whether the low risk in the general population—around 1–2%—is perceived as a meaningful risk for most people. The general population may also be less familiar with carrier screening services, or the potential benefits of these services. Chen and Goodson^50^ conducted a review of studies assessing factors that influence acceptance or decline of carrier screening for a single condition (CF) that identified similar issues.

Several studies have examined the public’s level of interest in expanded carrier screening. Some studies that included individuals of reproductive age have found general interest in expanded carrier screening.^35,36^ A survey of 777 Swedish parent couples^51^ found that about one-third of couples were interested in preconception carrier screening, just under one-third were not, and the remainder were uncertain. The authors attributed the range of interest in part to relatively low knowledge of carrier screening, which was neither standard of care nor marketed to consumers in Sweden. Common reasons for interest in this service include avoiding a future child’s suffering and preparation for a child with a serious disease.^35,36,51^ Common reasons for disinterest include opposition to carrier screening in principle, a lack of desire to know carrier status, and opposition to abortion.^35,51^ Two studies found that the majority of couples were willing to pay more than 75 € for the service.^35,36^

In addition, a growing number of studies are examining motivations among individuals who have actually received or declined to receive expanded carrier screening. A few studies have found that learning about and/or preparing for the possibility of a child with a genetic condition are key motivations for many of those who choose to get screening.^10,48,52,53^ Gilmore et al.^34^ surveyed 240 women planning a pregnancy who declined enrollment in a study involving genomics-based carrier screening and found the most common reasons for declining were lack of time or lack of interest in getting screening, with a smaller number indicating that they didn’t want to know the information or didn’t want to cause themselves anxiety. In another survey of pregnant women, Propst et al.^53^ found that those who declined screening felt the chance of finding something would be small, said the results wouldn’t change anything, or worried that the results would make them anxious. These data show that those who ultimately get or decline screening in a clinical setting are able to identify specific reasons for their decisions, suggesting that these are informed choices.

An important limitation of the existing data is that the vast majority of individuals included in these studies were European or of European ancestry and had a relatively high socioeconomic status and access to high-quality medical care. Thus, these data do not fully describe the public’s awareness, knowledge, and interest in this service. Further research is needed to understand how expanded carrier screening will be received among more diverse populations.

### Expanded carrier screening may not be a priority service for health-care systems

Health-care payers and delivery systems have limited resources and must choose among various options in determining allocation of those resources. A high priority is treatment of patients who are already sick, which could contribute to expanded carrier screening programs being given a low priority.^49^ Several have argued that the success of expanded carrier screening programs should be measured based on their ability to advance autonomous reproductive choice,^31,49^ rather than on the impact on birth prevalence and treatment costs of particular conditions. Because carrier screening needs to be a voluntary choice, uptake of screening is likewise a poor way to measure success of the program.

Expanded carrier screening does influence reproductive decisions for a high percentage of at-risk couples (i.e., both partners are carriers for the same autosomal recessive condition). A survey of 64 at-risk couples who received expanded carrier screening found three affected pregnancies, of which two were voluntarily terminated (carnitine palmitoyltransferase II deficiency [MIM 600650] and cystic fibrosis [MIM 219700]), and one was continued (cystic fibrosis).^54^ Among those couples who were pregnant (30%), the majority (53%) elected or planned a diagnostic procedure. Among those couples who were not pregnant, 62% planned to pursue prenatal alternative reproductive options or prenatal diagnosis. Nevertheless, “advancing autonomous reproductive choice” and voluntary services do not fit well within the traditional framework of clinical utility and medical necessity, which frequently drives system priorities and coverage decisions.^55^ In addition, expanded carrier screening programs are expected to benefit only a small proportion of the population, so health-care systems may not prioritize these services because of other health needs that could have greater impact among more people.^32^

Several studies of those considering and/or undergoing expanded carrier screening have suggested that screening has personal utility for many individuals; it may fulfill their curiosity or help them feel well prepared for the birth of a child.^48,56,57^ These studies point to a role of carrier screening beyond allowing increased reproductive options. However, this role does not support a justification for expanded carrier screening services on the basis of traditional clinical utility and medical necessity arguments. Moving toward a personal utility framework to support reimbursement can avoid framing expanded carrier screening as a tool to avoid the birth of individuals with specific genetic conditions, instead emphasizing the value of autonomous choice and reproductive control.

To the extent that expanded carrier screening is made available as a service through health-care delivery systems because of its perceived value, an essential obligation of program implementation is to ensure just and equitable access, including for all individuals who find expanded carrier screening personally useful regardless of their ability to afford out-of-pocket costs. While a program must be designed to ensure it provides accurate and balanced information and encourages autonomous decision making, a consideration for justice recognizes that access to screening is a necessary precondition for informed decision making to have value. There are numerous ways to address this concern such as through reimbursement strategy or by structuring the program as a national public health service.

### Expanded carrier screening programs have the potential to pressure participation

A potential concern about pan-ethnic carrier screening is that by offering carrier screening to everyone, it may not feel voluntary to decline to participate in the program.^17^ Routine use of expanded carrier screening may be unwelcome by some individuals who may feel pressured to undergo screening when it otherwise would not have been a priority for them.^31^ In the early 1990s, Bekker et al.^58^ demonstrated that the manner in which CF screening was offered influenced its uptake, which raised questions about whether and how screening should be offered or recommended. In addition, if there is decreased societal support for families with affected children, especially when resources and services are reduced, couples may not feel free to make a reproductive choice about whether to conceive an affected child.^17^

Evidence from high-risk populations provides support that at least some people may feel this pressure. Holtkamp^36^ surveyed Dutch Jewish individuals and found that 26% agreed or completely agreed with the statement “Offering a carrier test can cause people to feel forced to get tested,” and 24% agreed that “Healthcare professionals can force Jewish couples that want to have children to have a carrier test.” Other studies have shown a smaller degree of perceived pressure in the general population. Plantinga et al.^35^ surveyed Dutch couples and found that 10% of respondents agreed with the statement “I think that my partner and I as (future) parents have a responsibility to do this test,” and 3% agreed that “Carrier testing is socially expected from me and my partner.” Propst et al.^53^ found that the pregnant women they surveyed tended to disagree that they felt pressured to get expanded carrier screening.

Some options have been proposed to promote a voluntary decision, including providing varying levels of information depending on individual preference or incorporating minor barriers into the process, such as requiring a second health-care encounter. For example, some stakeholders have suggested that paying some amount might increase the deliberation about the decision to get screening.^49^ However, if the latter approach is used, there is also a risk of nonuniformly limiting access to screening, because it may be more difficult for some individuals, especially those who are traditionally underserved, to surmount those hurdles.

### Expanded carrier screening may reinforce disability-based discrimination, and may potentially be hurtful to people living with these conditions

Since the early days of carrier screening and prenatal genetic testing, there have been concerns about the potential for these programs to shape society’s attitudes about people with disabilities. The disability rights critique argues that screening programs have the potential to encourage the perception that the lives of individuals with certain genetic conditions are less valuable than those without such conditions.^59^ These concerns continue to pose challenges in the setting of expanded carrier screening. Some have proposed that a movement to genome or exome sequencing can diminish potential harms for individuals with disabilities by reducing the stigma associated with living with a condition labeled “severe.”^60,61^ Further research will be needed to understand how expanded carrier screening influences attitudes about disability.

### Genetic counseling needs to be adapted for expanded carrier screening services

Carrier screening programs have long confronted questions about what kind of counseling and informed consent should accompany screening. In focus groups with US genetics professionals, Cho et al.^62^ found major concerns about the limitations of expanded carrier screening, including false positives, ambiguous results, and potential misunderstanding of negative results. Participants in these groups highlighted the need for genetic counseling to overcome the challenges of informed consent and results interpretation.^63^ More recently, Janssens et al.^32^ interviewed 16 European geneticists on their views on pre- and posttest counseling for expanded carrier screening. They reported that one of the main challenges to pretest counseling was enabling informed decisions about the conditions to be screened. They noted that it is impractical for parents to consent to specific disorders, and a simplified approach is needed, such as offering categories of conditions based on common characteristics.^32,45,64,65^ Similar concerns have also been identified in a survey of North American genetic counselors (*n* = 377)^27^ and interviews with Dutch stakeholders (*n* = 17), who also commented that there is a need for better infrastructure, guidelines, education, and counseling tools.^17,49^

From the patient and public perspective, there is evidence that some individuals experience confusion about expanded carrier screening. Rothwell et al.^10^ interviewed 17 prenatal patients at a university hospital who had received positive carrier results and found that some were confused about the difference between carrier screening and prenatal testing, as well as emotional uncertainty due to variation in how results were discussed. Kraft et al.^66^ similarly found some confusion between carrier screening and prenatal testing among preconception women and partners with negative carrier screening results, although on the whole their findings suggested that there were few psychosocial harms to those who received negative results. In that study, individuals who received expanded, genomics-based screening neither used more health-care services nor experienced more anxiety and depression than did those who received usual care.^67^ These concerns align with the results of a survey of 272 Australian students in Jewish high schools showing that those who received an expanded screening panel had lower knowledge levels than those who had single-disease screening.^68^ Taken together, these data suggest that expanded carrier screening may require the development of novel tools to support shared decision making and adequate understanding of results, but that concerns about psychosocial harms may be overstated.

### Technical challenges of the knowledge base and newer sequencing platforms

There are limitations to current knowledge and sequencing technologies that could constrain the value of expanded carrier screening for some conditions or populations. For example, some important genes for carrier screening are technically challenging to sequence because of large structural alterations or pseudogenes (e.g., *SMN1* [MIM *600354] for spinal muscular atrophy). In addition, our current knowledge base and ability to interpret sequence variants is continuing to evolve, and many variants have unknown pathogenicity. The chances of detecting variants of unknown significance may vary depending on racial and ethnic group. For instance, common pathogenic variants for CF that are found in non-Hispanic Caucasian populations do not account for a large proportion of pathogenic variants in other groups, with a range of 48% among Asian Americans to 94% among Ashkenazi Jewish individuals.^69,70^ Expanded carrier screening using sequence analysis rather than targeted analysis of predefined variants may identify many novel sequence variants with unknown significance, and this is particularly likely among populations that have been less well studied in the past. Finally, many conditions have variable phenotypic expression or penetrance (e.g., *GBA* [MIM *606463] associated with Gaucher disease), even for individuals within a family segregating the same known variants, so it may be difficult to accurately predict the disease course or phenotype based on genotype alone. For these conditions, there may be limited value in disclosing carrier status for reproductive decision making.

### Emerging challenges

Expanded carrier screening exists in a broader technological, social, and political context that will continue to pose new challenges. This service is part of a complex network of genetic technologies.^71^ For example, noninvasive prenatal testing and newborn screening can detect genetic conditions, but as illustrated by several empirical studies,^10,66^ individuals may struggle to distinguish between these services. Additionally, the potential for new gene-editing techniques offers the possibility of expanding opportunities to use the information that carrier screening detects.

Further, sociopolitical debate about reproductive rights and access to (including payment for) health care will have major effects on how carrier screening is used in practice. As some researchers have noted,^72^ views on issues such as distribution of resources and reproductive rights must be interpreted in the context of the stakeholders’ sociopolitical context. For example, the largely private, individual-based health insurance structure in the United States creates distinct financial incentives and considerations for stakeholders, in contrast to countries with nationalized health-care systems. Likewise, laws and social attitudes regarding abortion, as well as the availability and accessibility of advanced reproductive technologies, are likely to influence stakeholder attitudes, clinical utility, and personal utility.

## Conclusion

Expanded carrier screening programs that increase the number of conditions screened and expand to pan-ethnic populations hold significant promise in providing more equitable access to carrier screening services, maximizing autonomous reproductive choice, reducing the potential for stigma associated with being a carrier, and improving efficiency and cost-effectiveness of the service. While commercial expanded carrier screening is currently available, as recently as 2015 most clinicians were not yet offering this service to their patients and only a small minority of pregnant individuals received this service in the United States.

There are significant challenges to successful implementation of expanded carrier screening programs. The general population has a lack of familiarity with and understanding of genetic conditions, may not perceive themselves to be at risk of being a carrier, and may have limited perceived benefit of expanded carrier screening services. Among medical experts and in the general population, there is disagreement about who should be offered these services or what conditions should be included as part of an expanded carrier screening program. Health systems may consider these services to have low priority. The genetics community will need to address practical challenges associated with pre- and posttest counseling. Expanded carrier screening programs will need to ensure that they are implemented in such a way that participation is voluntary, and that the program does not lead to disability-based discrimination. Finally, the evolving technological and sociopolitical context of these programs will continue to pose new challenges for clinical implementation.

Despite these challenges, carrier screening programs have historically overcome these barriers for individual or small panels of conditions, and there remains a significant potential benefit to expanding carrier screening services. The lessons from existing programs can be leveraged to overcome these challenges to expanding carrier screening in the future.
